# A narrative review of mutant isocitrate dehydrogenase AML in Japan based on experience with ivosidenib in AGILE

**DOI:** 10.1007/s12185-026-04175-5

**Published:** 2026-03-03

**Authors:** Yasushi Hiramatsu, Shingo Urata, Takayuki Ishikawa, Takahiro Yamauchi

**Affiliations:** 1 Japanese Red Cross Society Himeji Hospital, Himeji, Japan; 2 Matsuyama Red Cross Hospital, Matsuyama, Japan; 3 Kobe City Medical Center General Hospital, Kobe, Japan; 4 Present Address: Takanohara Central Hospital, Nara, Japan; 5 University of Fukui Hospital, Fukui, Japan

**Keywords:** Acute myeloid leukemia, Ivosidenib, Isocitrate dehydrogenase

## Abstract

Ivosidenib is a mutant IDH1 (mIDH1) inhibitor that demonstrated clinical benefit in combination with azacitidine for treatment of m*IDH1* acute myeloid leukemia (AML) in the phase 3 AGILE trial. Results from AGILE led to the approval of ivosidenib plus azacitidine for patients with newly diagnosed m*IDH1* AML unfit for intensive chemotherapy in Japan. However, data on the use of ivosidenib plus azacitidine in Japanese patients with m*IDH1* AML are lacking. Here we present data from six Japanese patients enrolled in AGILE, of whom, three received ivosidenib plus azacitidine and three received placebo plus azacitidine. At data cut-off, Japanese patients in AGILE had an overall survival of 4.4–12.8 months and 24.8–36.5 months in the placebo plus azacitidine and ivosidenib plus azacitidine groups, respectively. Of the Japanese patients enrolled in AGILE, 2 of 3 (66.7%) patients receiving ivosidenib plus azacitidine and none receiving placebo plus azacitidine achieved complete remission within 24 weeks. Results from the Japanese population enrolled in AGILE are aligned with those of the overall study. The efficacy of ivosidenib plus azacitidine in Japanese m*IDH1* AML patients enrolled in AGILE who were ineligible for intensive chemotherapy appears to be consistent with the overall study population.

## Introduction

In Japan, the annual incidence of acute myeloid leukemia (AML) is increasing with the aging population [1]. Patients < 70 years of age have an annual incidence of AML of 0.6–6.0 per 100,000 in Japan, compared to 10–17 per 100,000 in patients ≥ 70 years of age [1]. The 5-year mortality rate of Japanese patients with AML is 34% in females and 28% in males [2]. In a multicenter, prospective observational study of AML in Japan, 42% of patients were younger than 65 years at diagnosis [2].

Currently in Japan, the recommended treatment for patients with AML is cytarabine plus an anthracycline [3]. However, some patients may be ineligible for intensive chemotherapy due to age, performance status, or comorbidities. The recommendation for Japanese patients with AML that are ineligible for intensive chemotherapy is treatment with venetoclax + azacitidine, venetoclax + low-dose cytarabine (LDAC), reduced intensity therapy or best supportive care (BSC) [3]. On March 27, 2025, ivosidenib, a mutant isocitrate dehydrogenase 1 (mIDH1) inhibitor, in combination with azacitidine, was approved for the treatment of patients with m*IDH1* AML that are ineligible for intensive chemotherapy [4].

## Isocitrate dehydrogenase 1 mutations in AML

The IDH1 enzyme catalyzes the oxidative decarboxylation of isocitrate, producing α-ketoglutarate (α-KG) [5]. Gain-of-function mutations in *IDH1* lead to the production of mIDH1, which converts α-KG to 2-hydroxyglutarate (2-HG) [5]. The resulting accumulation of 2-HG leads to widespread effects on cellular metabolism and epigenetics, which drives oncogenesis in m*IDH1* AML [5]. Mutations in *IDH1* have been shown to be present in 6–10% of patients with AML [6]. The impact of m*IDH1* on prognosis in AML remains unclear, however, generally patients with m*IDH1* have poorer survival outcomes than those with *IDH1* wild type AML [7].

Ivosidenib demonstrated encouraging efficacy in combination with azacitidine for the treatment of patients with newly diagnosed m*IDH1* AML unfit for intensive chemotherapy in the double-blind, randomized, placebo-controlled, phase 3 AGILE trial [8]. AGILE was a multicenter, multinational study that included patients from North America, South America, Europe and Asia, including Japan [8]. The primary endpoint of AGILE was event-free survival, which was defined as the time from randomization until treatment failure, relapse from remission, or death from any cause, whichever occurred first [8]. The results of AGILE demonstrated that ivosidenib + azacitidine had clinical benefit and was well-tolerated in patients with m*IDH1* AML [8]. A long-term analysis of AGILE showed that, at a median follow-up of 28.6 months, median overall survival (OS) was longer in the ivosidenib + azacitidine group than the placebo + azacitidine (29.3 months vs 7.9 months; HR: 0.42 (95% confidence interval [CI] 0.27–0.65); *P* < 0.0001) [9].

Ivosidenib is approved for the treatment of newly diagnosed m*IDH1* AML, in combination with azacitidine, by the European Medicines Agency (EMA) and US Food and Drug Administration (FDA) based on the clinical benefit demonstrated in AGILE [10, 11]. As of March 27, 2025, ivosidenib, in combination with azacitidine, is approved by the Pharmaceuticals and Medical Devices Agency (PMDA) for the treatment of patients with newly diagnosed m*IDH1* AML in Japan [4].

## Epidemiology of *IDH1* mutations in Japan

Data are limited on the frequency of *IDH1* mutations in Japan, which highlights an unmet need for further investigation. In a prospective genomic profiling study in Japan, 7.8% of patients with newly diagnosed or relapsed/refractory AML that were ineligible for intensive chemotherapy had m*IDH1* [12]*.* In the Japanese population of the VIALE-C which investigated the use of venetoclax in combination with LDAC in patients ineligible for intensive chemotherapy, 22.2% and 18.8% of patients had *IDH1/2* mutations in the placebo + LDAC and venetoclax + LDAC treatment groups, respectively [13]. In VIALE-A, which investigated the use of venetoclax + azacitidine for treatment of patients with newly diagnosed AML ineligible for intensive chemotherapy, 28.6% and 30.8% of Japanese patients had *IDH1/2* mutations in the venetoclax + azacitidine and placebo + azacitidine groups, respectively [14]. It has been shown that Japanese patients with AML with *IDH* mutations have reduced 5-year OS rate compared to those without *IDH* mutations, regardless of *NPM1* mutational status [15]. Previous data from the USA have shown that the frequency of *IDH1* mutations in AML increases with age, however there are no equivalent data from Japan [16]. The incidence and trends in hematological malignancies are broadly similar between Japan and the USA, so it can be inferred that the incidence of *IDH1* mutations will also increase with age in the Japanese population [17].

These findings underscore the importance of routine genomic profiling in Japan, particularly as targeted therapies, such as mIDH1 inhibitors, become available. The use of genetic testing for prognostic stratification, determination of transplant eligibility, and treatment selection is recommended by the Japanese Society of Hematology [3]. Despite this, the use of genomic testing to identify potential mutational targets is limited in Japan. Therefore, there is currently a missed opportunity for use of targeted agents, such as mIDH inhibitors, in Japan [18]. Overall, there is a lack of recommended treatment options available for patients with AML ineligible for intensive chemotherapy in Japan and this patient population have poor survival outcomes.

## Current treatment of m*IDH* AML in Japan

Currently, Japanese treatment guidelines recommend venetoclax + azacitidine, venetoclax + LDAC, reduced-intensity therapy, or BSC for the treatment of patients with AML that are ineligible for intensive chemotherapy [3]. However, in the real-world, cytarabine, aclarubicin ± granulocyte colony-stimulating factor and azacitidine have been used as treatment options for this patient population in Japan [19–21]. A real-world study in Japan showed that median OS of patients with AML that are ineligible for intensive chemotherapy, ranges from 2.2 months for patients receiving LDAC and BSC, to 9.2 months for patients receiving azacitidine [22]. In the VIALE-A trial, among patients with m*IDH1/2* AML, OS at 12 months was 66.8% in the venetoclax + azacitidine group and 35.7% in the placebo + azacitidine group (*P* < 0.001) [23]. Taken together, these data show that there is a population of patients that are ineligible for intensive chemotherapy with m*IDH1* AML in Japan who are not receiving appropriate targeted treatment.

The use of mIDH inhibitors, such as the newly approved ivosidenib (in combination with azacitidine), provides an avenue for treatment for patients that are ineligible for intensive chemotherapy. Despite its recent approval by the PMDA, no studies investigating the use of ivosidenib + azacitidine in patients that are ineligible for intensive chemotherapy have been performed in Japan. Given the poor outcomes of this patient population and the recent approval of ivosidenib in Japan, it is clinically important to assess the efficacy of ivosidenib in Japanese patients. Thus, below we discuss data from the six Japanese patients included in the AGILE study.

## Use of ivosidenib + azacitidine in Japanese population

In Japan, 113 patients were screened for eligibility for AGILE. Nine patients were determined to have m*IDH1* AML based on central testing, three of which were excluded due to screening failures. The remaining six patients with m*IDH1* AML were enrolled in AGILE from four centers in Japan, of which three received ivosidenib + azacitidine and three received placebo + azacitidine. Due to the small number of patients in the Japanese subset, data from these patients cannot be directly compared to results from the AGILE trial population or other studies. However, the results of the Japanese subset of AGILE are discussed below in the context of results from other studies.

### Patient characteristics

The baseline characteristics of Japanese patients included in AGILE are described in Table 1. Of these patients, 5/6 patients (83.3%) did not have *IDH1* mutational testing data available at a local level and instead underwent *IDH1* mutation testing centrally at inclusion of the study **(**Table 1**)**. This appears to confirm the lack of widespread mutational testing observed in Japan.

**Table 1:** Baseline characteristics of Japanese patients in AGILE

	Ivosidenib + azacitidine (N = 3)		Placebo + azacitidine (N = 3)
Age, mean (individual values), years	74.3 (77, 84, 62)		72.3 (72, 76, 69)
Male, n (%)	2 (66.7)		1 (33.3)
BMI, mean (individual values), kg/m^2^	22.9 (26.1, 16.6, 25.9)		23.6 (22.9, 27.1, 20.8)
*Nature of AML per IWRS, n (%)*			
De novo	3 (100.0)		2 (66.7)
Secondary	0 (0.0)		1 (33.3)
*ECOG performance status, n (%)*			
0	2 (66.7)		1 (33.3)
1	1 (33.3)		1 (33.3)
2	0 (0.0)		1 (33.3)
*Cytogenetic risk status by investigator, n (%)*			
Intermediate	1 (33.3)		2 (66.7)
Poor	2 (66.7)		1 (33.3)
Bone marrow aspirate blasts, individual values, %	44; 53; 58		46; 67; 94
Peripheral blood blasts, individual values, %	4.0; 26.0; 29.0		8.0; 34.0; 96.5
*IDH1 mutation type based on central testing, n (%)*			
R132C	1 (33.3)		0 (0.0)
R132G	0 (0.0)		1 (33.3)
R132H	1 (33.3)		0 (0.0)
R132S	1 (33.3)	2 (66.7)	

BMI, body mass index. ECOG PS, Eastern Cooperative Oncology Group performance status; *IDH1,* isocitrate dehydrogenase 1; IWRS, interactive web response system

In the Japanese subset of patients from AGILE, 50.0% and 33.3% of patients with m*IDH1* AML had an Eastern Cooperative Oncology Group performance status (ECOG PS) of 0 and 1, respectively. This suggests this small Japanese subset have better performance status than the total AGILE population (16.4% had an ECOG PS of 0) [8]. By comparison, in the VIALE-C study of Japanese patients that were ineligible for intensive chemotherapy, 27.8% and 33.3% had an ECOG PS of 0 in the placebo + LDAC and venetoclax + LDAC groups, respectively [13]. The proportion of Japanese patients enrolled in VIALE-A with ECOG PS 0 and 1 was not reported [14]. In a real-world study of patients that were ineligible for intensive chemotherapy in Japan, the percentage of patients with an ECOG PS of 0–1 ranged from 31% in patients treated with BSC to 70% in patients treated with azacitidine [22]. This shows that the ECOG PS of Japanese patients from AGILE is typical of Japanese patients that are ineligible for intensive chemotherapy in the real-world.

In the Japanese subset of patients from AGILE, 50.0% of patients had a R132S m*IDH1* mutation **(**Table 1**)**. Whereas, in the AGILE trial population, the R132S m*IDH1* was relatively rare (present in 3% and 8% of patients in the ivosidenib + azacitidine and placebo + azacitidine groups, respectively) [8]. In a Japanese real-world observational study, only 0.4% (1/233) of patients had a R132S *IDH1* mutation [15]. However, further study is needed in a larger population to determine the frequency of R132S *IDH1* mutations in Japanese patients that are ineligible for intensive chemotherapy.

### Outcomes

**Fig. 1:**
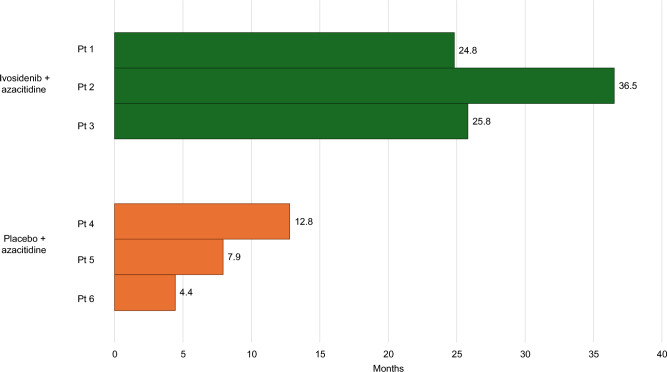
Overall survival of individual Japanese patients included in AGILE at data cut-off. Data cut-off date of June 30, 2022. Pt, patient

In AGILE, Japanese patients treated with placebo + azacitidine had an OS of 4.4–12.8 months, and Japanese patients treated with ivosidenib + azacitidine had a longer OS of 24.8–36.5 months, using a data cut-off date of June 30, 2022 **(**Fig. 1**)**. Two of the three Japanese patients receiving ivosidenib + azacitidine were alive at this data cut-off. In a long-term analysis of the AGILE trial population, the median OS was 29.3 (95% CI 13.2-not reached) months in the ivosidenib + azacitidine group and 7.9 (95% CI 4.1–11.3) months in the placebo + azacitidine group [9]. These median values are similar to the OS values observed in the Japanese subset of AGILE. The real-world OS of patients with AML that are ineligible for intensive induction chemotherapy in Japan (5.4 [95% CI 2.7–14.6] months) is lower than Japanese patients treated with ivosidenib + azacitidine in AGILE [22]. Interestingly, the Japanese subgroup of the VIALE-C trial receiving venetoclax + LDAC had a shorter median OS than patients receiving placebo + LDAC (4.7 versus 8.1 months), however these results may be due to the small size of this patient population [13]. Conversely, in VIALE-A, for Japanese patients ineligible for intensive chemotherapy, median OS was not reached in the venetoclax + azacitidine group and was 8.6 months in the placebo + azacitidine group, at a median follow-up of 16.3 months [14].

**Fig. 2:**
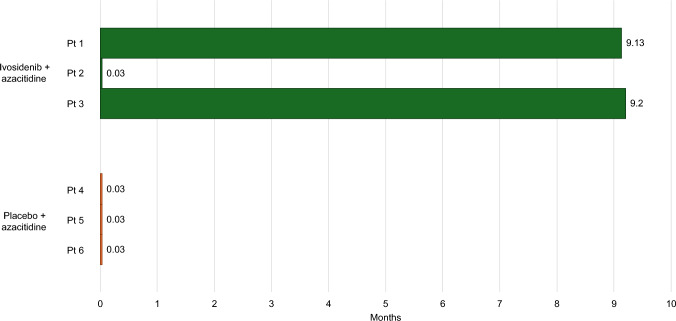
Event-free survival of individual Japanese patients included in AGILE at data cut-off. Data cut-off date of March 18, 2021. Event-free survival was defined as the time from randomization until treatment failure (i.e., the patient did not achieve complete remission by week 24), relapse, or death from any cause, whichever occurred first. Patients who did not achieve complete remission by week 24 were considered to have had an event at the date of randomization (0.03 months). At data cut-off (March 18, 2021), all patients in the ivosidenib + azacitidine were still enrolled in the study and all patients in the placebo + azacitidine group had died. Pt, patient

In AGILE, the event-free survival was defined as the time from randomization until treatment failure (i.e., the patient did not have complete remission [CR] by week 24), relapse, or death from any cause, whichever occurred first [8]. Patients who did not have CR by week 24 were considered to have had an event at the date of randomization (i.e., 0.03 months). In the Japanese subset of AGILE, the event-free survival (using a data cut-off date of March 18, 2021) in the ivosidenib + azacitidine group ranged from 0.03 to 9.20 months **(**Fig. 2**)**. All patients in the placebo + azacitidine group had an event-free survival of 0.03 months **(**Fig. 2**)**. In the AGILE trial population, as more than half of patients did not have CR by week 24, median event-free survival was the same in both treatment groups (0.03 months) [8]. However, in an exploratory analysis which defined treatment failure as a lack of CR, CR with incomplete hematologic recovery, or morphologic clearance of leukemic cells from the marrow after at least 24 weeks of treatment, event-free survival was 22.9 months in the ivosidenib + azacitidine group and 4.1 months in the placebo + azacitidine group [8]. Event-free survival was longer in patients receiving ivosidenib + azacitidine compared to those treated with placebo + azacitidine in the Japanese population of AGILE.

At data cut-off of March 18, 2021, of the Japanese patients enrolled in AGILE, two patients achieved CR and one patient achieved a morphologic leukemia-free state in the ivosidenib + azacitidine group. Whereas, no patients achieved CR in the placebo + azacitidine group—two patients had stable disease and one patient was not evaluable. In the AGILE trial population, CR (within 24 weeks) occurred in 38% of patients in the ivosidenib + azacitidine group and 11% of patients in the placebo + azacitidine group [8]. Thus, the increased occurrence of CR in the ivosidenib + azacitidine group observed in the AGILE trial was also demonstrated in the Japanese subset of patients. The duration of CR was 5.5 and 5.6 months in Japanese patients receiving ivosidenib + azacitidine. The median duration of response in the AGILE trial was 22.1 months (95% CI 13.0-not evaluable) in the ivosidenib + azacitidine group and 9.2 months (95% CI 6.6–14.1) in the placebo + azacitidine group [8]. While the duration of response in the Japanese patients enrolled in AGILE was shorter than that of the overall study population, the data cut-off date used for this analysis was earlier than the date used for OS analysis. Therefore, comparison of OS values between the Japanese subset and overall AGILE population may be more informative.

Three patients provided samples for *IDH1* mutation clearance analysis (one in the ivosidenib + azacitidine group, and two in the placebo + azacitidine group). The patient receiving ivosidenib + azacitidine cleared the *IDH1* mutation from both the peripheral blood and bone marrow mononuclear cells. Whereas patients receiving placebo + azacitidine did not clear the mutation from either cell type.

### Safety

The Japanese patients in AGILE were exposed to treatment for 113.0–333.0 days in the ivosidenib + azacitidine group and 19.0–327.0 days in the placebo + azacitidine group. The relative dose intensity of ivosidenib or placebo was 47.0–99.3% and 92.7–100.0% in the ivosidenib + azacitidine and placebo + azacitidine groups, respectively.

Safety outcomes of Japanese patients in AGILE are described in Table 2. All patients in the Japanese subset of AGILE experienced grade ≥ 3 treatment-emergent adverse events (TEAEs). This is similar to the AGILE trial population, in which 93% and 95% of patients experienced grade ≥ 3 TEAEs in the ivosidenib + azacitidine and placebo + azacitidine groups, respectively [8]. In the Japanese subset of AGILE, the most frequent grade ≥ 3 TEAE was febrile neutropenia in the ivosidenib + azacitidine group and anemia in the placebo + azacitidine group **(**Table 2**)**. Similarly, in the AGILE population, the most frequent TEAEs were febrile neutropenia and anemia, demonstrating that the safety profile in the Japanese subset of AGILE was generally consistent with the safety profile observed in the AGILE population as a whole [8]. In the AGILE trial population, 14% of patients receiving ivosidenib + azacitidine developed differentiation syndrome of any grade [8]. One patient receiving ivosidenib had differentiation syndrome (two events) in the Japanese subset of AGILE, one event was grade ≥ 3. This grade 3 differentiation syndrome was managed by dexamethasone, oxygen support, and temporary interruption of ivosidenib treatment. The mild differentiation syndrome event was managed with dexamethasone. Both episodes of differentiation syndrome resolved and the patient continued with ivosidenib treatment following stabilization.

**Table 2:** Safety outcomes of Japanese patients in AGILE

	Ivosidenib + azacitidine (N = 3)	Placebo + azacitidine (N = 3)
*Grade ≥ 3 treatment-emergent adverse events, n (%)*		
Any	3 (100.0)	3 (100.0)
Febrile neutropenia	2 (66.7)	1 (33.3)
Anemia	1 (33.3)	3 (100.0)
Anaphylactic reaction	1 (33.3)	0 (0.0)
Decrease appetite	1 (33.3)	1 (33.3)
Differentiation syndrome	1 (33.3)	0 (0.0)
Drug eruption	1 (33.3)	0 (0.0)
Electrocardiogram QT prolonged	1 (33.3)	0 (0.0)
Pneumonia	1 (33.3)	1 (33.3)
Bacteremia	0 (0.0)	1 (33.3)
C-reactive protein increased	0 (0.0)	1 (33.3)
Disseminated intravascular coagulation	0 (0.0)	1 (33.3)
Hypophosphatemia	0 (0.0)	1 (33.3)
Thrombocytopenia	0 (0.0)	1 (33.3)

One patient receiving ivosidenib + azacitidine had nausea and prolonged QT interval on an electrocardiogram (grade ≥ 3) that were deemed to be related to treatment with ivosidenib. However, this patient received concomitant treatment with antifungal agents. Despite this, no Japanese patients in AGILE discontinued ivosidenib due to TEAEs. However, one patient in the ivosidenib + azacitidine group discontinued azacitidine due to febrile neutropenia.

## Conclusions

The efficacy and safety of ivosidenib for the treatment of patients with m*IDH1* AML who are ineligible for intensive chemotherapy was demonstrated in the AGILE clinical trial. However, there are limited real-world data available for patients with m*IDH1* AML that are ineligible for intensive chemotherapy in Japan, perhaps due to a lack of mutational testing in routine clinical practice. The data presented here from the Japanese patients enrolled in AGILE provides valuable evidence that treatment with ivosidenib has clinical benefit in this population. Overall, these results highlight the importance of routine *IDH1* testing in Japan and support the clinical relevance of ivosidenib, in combination with azacitidine, as a therapeutic option for Japanese patients with AML that are ineligible for intensive chemotherapy. As targeted agents become increasingly integrated into AML treatment pathways, these results may guide clinical decision-making for this molecularly distinct population.

## Data Availability

De-identified patient- and/or study-level clinical trial data, including the clinical study report and study protocol, will be shared, in line with the Servier Data-Sharing Policy (available at https://vivli.org/ourmember/servier/).
